# Structural insights into auxin recognition and efflux by *Arabidopsis* PIN1

**DOI:** 10.1038/s41586-022-05143-9

**Published:** 2022-08-02

**Authors:** Zhisen Yang, Jing Xia, Jingjing Hong, Chenxi Zhang, Hong Wei, Wei Ying, Chunqiao Sun, Lianghanxiao Sun, Yanbo Mao, Yongxiang Gao, Shutang Tan, Jiří Friml, Dianfan Li, Xin Liu, Linfeng Sun

**Affiliations:** 1 The First Affiliated Hospital of USTC, MOE Key Laboratory for Membraneless Organelles and Cellular Dynamics, Hefei National Laboratory for Physical Sciences at the Microscale, School of Life Sciences, Division of Life Sciences and Medicine, University of Science and Technology of China, Hefei, China; 2 CAS Center for Excellence in Molecular Cell Science, Shanghai Institute of Biochemistry and Cell Biology, University of CAS, Chinese Academy of Sciences (CAS), Shanghai, China; 3 MOE Key Laboratory for Membraneless Organelles and Cellular Dynamics, Hefei National Laboratory for Physical Sciences at the Microscale, Division of Life Sciences and Medicine, University of Science and Technology of China, Hefei, China; 4 Cryo-EM Center, Core Facility Center for Life Sciences, University of Science and Technology of China, Hefei, China; 5 Institute of Science and Technology Austria (IST Austria), Klosterneuburg, Austria; 6 Biomedical Sciences and Health Laboratory of Anhui Province, University of Science and Technology of China, Hefei, China

**Keywords:** Cryoelectron microscopy, Membrane proteins, Auxin

## Abstract

Polar auxin transport is unique to plants and coordinates their growth and development^1,2^. The PIN-FORMED (PIN) auxin transporters exhibit highly asymmetrical localizations at the plasma membrane and drive polar auxin transport^3,4^; however, their structures and transport mechanisms remain largely unknown. Here, we report three inward-facing conformation structures of *Arabidopsis thaliana* PIN1: the apo state, bound to the natural auxin indole-3-acetic acid (IAA), and in complex with the polar auxin transport inhibitor *N*-1-naphthylphthalamic acid (NPA). The transmembrane domain of PIN1 shares a conserved NhaA fold^5^. In the substrate-bound structure, IAA is coordinated by both hydrophobic stacking and hydrogen bonding. NPA competes with IAA for the same site at the intracellular pocket, but with a much higher affinity. These findings inform our understanding of the substrate recognition and transport mechanisms of PINs and set up a framework for future research on directional auxin transport, one of the most crucial processes underlying plant development.

## Abstract (web summary)

Structures of the *Arabidopsis thaliana* auxin exporter PIN1 in the apo state, bound to the natural auxin or bound to an inhibitor provide insights into the polar auxin transport mechanisms mediated by PIN family transporters.

## Main

**Extended Data Fig. 1:**
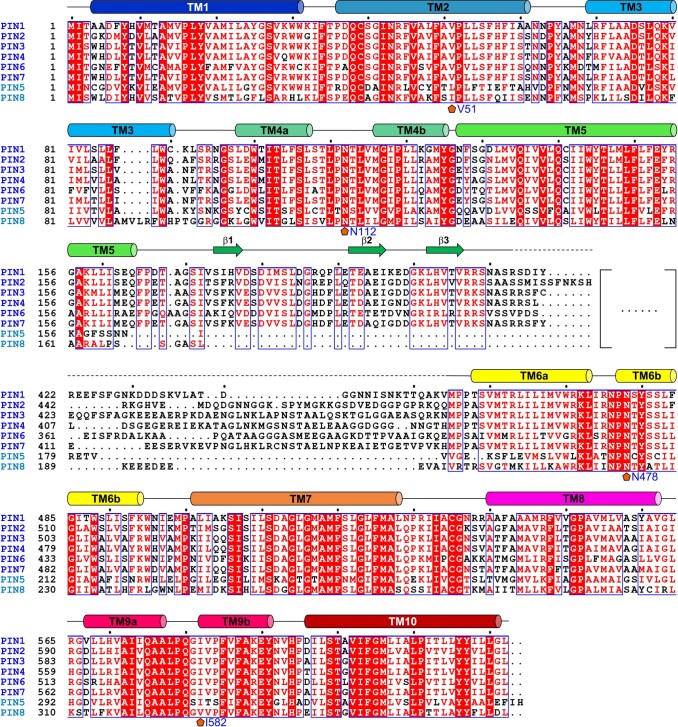
Sequence alignments of 8 PINs from *Arabidopsis thaliana*. Sequences for the 8 PINs are available at Uniprot (https://www.uniprot.org) with the following accession codes: PIN1: Q9C6B8, PIN2: Q9LU77, PIN3: Q9S7Z8, PIN4: Q8RWZ6, PIN5: Q9FFD0, PIN6: Q9SQH6, PIN7: Q940Y5, PIN8: Q9LFP6. Sequences are aligned using ClustalW. Secondary structural elements of PIN1 are indicated above the sequence. Invariant amino acids are shaded red. The IAA interacting residues are indicated by orange pentagons.

Auxin has a pivotal role in regulating almost every aspect of plant growth and development^2,6^. A distinguishing feature of auxin is its directional cell-to-cell transport—polar auxin transport—which enables plants to coordinate development and adapt to exogenous signals^1^. PIN auxin exporters^3,4^ are critical to this process and have received considerable attention, as their polar subcellular localizations determine the directionality of polar auxin transport and have key roles in the asymmetric distribution of auxin and plant development^7–9^. In *A. thaliana*, PIN1 to PIN8 share two conserved domains, the N-terminal and C-terminal domains, joined by a less conserved hydrophilic loop^10^ (Extended Data Fig. 1). On the basis of the length of the hydrophilic loop, PINs can be classified as long (PIN1–PIN4 and PIN7), short PINs (PIN5 and PIN8) and intermediate^8^ (PIN6). The short PINs are typically found at the endoplasmic reticulum, whereas the long PINs are at the plasma membrane and dynamically regulated by endocytosis and recycling^8,11–14^. NPA, an inhibitor of auxin export and polar auxin transport, has been instrumental in establishing a key role of auxin efflux in polar auxin transport. NPA directly targets PINs^15,16^, but its mechanism of action remains unknown.

*Arabidopsis* PIN1 and PIN2 (also known as EIR1) were the first PINs to be identified^17,18^. *Arabidopsis* PIN1 is widely expressed, and has crucial roles in embryos, apical meristems and vascular tissues, as well as in developing organs^8,19^. *pin1* deficiency results in naked, pin-shaped inflorescences^9^. In this study, we set out to determine the structure of PIN1 and reveal the basis of substrate recognition and the mechanism of inhibition by NPA.

## PIN1-mediated auxin efflux in HEK293F cells

**Fig. 1:**
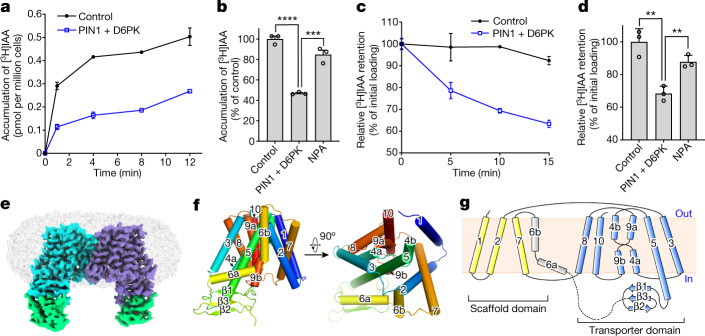
Characterization of PIN1-mediated auxin transport in HEK293F cells and the cryo-EM structure of dimeric PIN1. **a**, Loading assay showing that accumulation of [^3^H]IAA is decreased in cells co-expressing PIN1 and D6PK compared with control cells expressing D6PK only. **b**, Relative [^3^H]IAA accumulation in the absence or presence of NPA measured at 5 min. **c**, Efflux assay showing that [^3^H]IAA retention is decreased in cells co-expressing PIN1 and D6PK compared with control cells. **d**, [^3^H]IAA retention relative to the initial loading in the absence or presence of NPA, measured at 10 min. Three independent experiments performed for each point or construct in **a**–**d**. Two-tailed unpaired *t*-test. **P = 0.0041 for control versus PIN1 plus D6PK, **P = 0.0052 for NPA in with NPA versus no NPA, ***P = 0.001, **** *P* < 0.0001. Data are mean ± s.d. **e**, Overview of the electron density for PIN1. Densities corresponding to the PIN1 protomers are coloured cyan and purple, respectively. The sybodies are coloured green. **f**, Overall structure of a PIN1 monomer. **g**, A topology diagram of PIN1. Source data.

**Extended Data Fig. 2:**
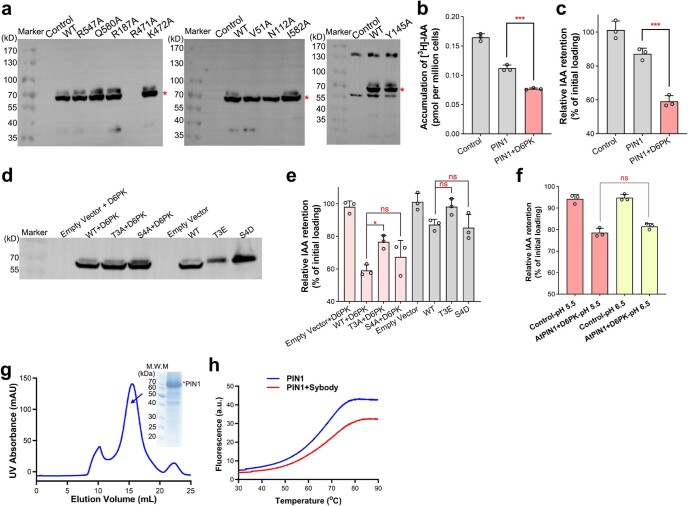
PIN1-mediated auxin transport in HEK293F cells. **a**, PIN1 expression detected by whole-cell western blot using anti-Flag tag antibody. Independent experiments have been repeated for three times with similar results. **b**, Co-expression of PIN1 and D6PK further decreases [^3^H]-IAA accumulation in loading assay. **c**, Co-expression of PIN1 and D6PK further decreases [^3^H]-IAA retention in auxin efflux assay. Independent experiments were repeated three times for constructs in **(b**, **c)**. Significances were determined using a two-tailed unpaired *t*-test. *** *P* = 0.0004 for *At*PIN1 versus PIN1 plus D6PK in **(b)**, *** *P* = 0.0005 for PIN1 versus PIN1 plus D6PK in **(c)**. Data are presented as mean ± SD. **d**, Expressions of the PIN1 phosphosites mutants examined by western blot. T3E mutant had a relatively decreased expression while all other constructs have similar expression levels. **e**, Relative [^3^H]-IAA retention for the phosphosites mutants in efflux assay. Independent experiments were repeated three times for each construct. Significances were determined using a two-tailed unpaired *t*-test. * *P* = 0.0182, ns = not significant. Data are presented as mean ± SD. **f**, IAA efflux at different pH. After [^3^H]-IAA loading in pH 5.5 buffer, cells were transferred to a buffer with a pH of 5.5 or 6.5, respectively. n = 3 biologically independent experiments for each group. Significances were determined using a two-tailed unpaired *t*-test. ns = not significant. Data are presented as mean ± SD. **g**, Representative gel filtration and Coomassie-blue-staining SDS-PAGE results of WT PIN1 alone. Independent experiments have been repeated for three times with similar results. **h**, Microscale fluorescent based thermal stability assay result for the PIN1 protein alone, or with Sybody-21. The thermal stability of PIN1 was increased by ~5 degrees in the presence of Sybody-21. Full version of all gels and blots are provided in Supplementary Fig. 1. Source data

Auxin efflux activity of PIN has been demonstrated using *Arabidopsis* protoplasts, tobacco cultured cells, yeast, human HeLa cells and *Xenopus* oocytes^3,14^. Notably, in oocytes, auxin efflux activity was detected only when PIN was co-expressed with a kinase such as D6 PROTEIN KINASE^20^ (D6PK). We expressed *A. thaliana* PIN1 in HEK293F cells (Extended Data Fig. 2a) and characterized its transport activity. In the loading assay, cells co-expressing PIN1 and D6PK accumulated much less radioisotope-labelled [^3^H]IAA compared with cells transfected with D6PK alone (Fig. 1a,b). In the efflux assay, which measures radiolabelled auxin over time after transfer into isotope-free buffer, residual [^3^H]IAA remained almost unchanged in the control condition but was markedly decreased in cells co-expressing PIN1 and D6PK (Fig. 1c,d). Notably, expressing PIN1 alone resulted in accumulation of [^3^H]IAA in the loading and efflux assays compared with control cells, but less than in cells co-expressing D6PK (Extended Data Fig. 2b,c). These results show that PIN1 mediates active auxin efflux when expressed in HEK293F cells, and is further activated by D6PK. This PIN1-mediated auxin efflux is inhibited by NPA (Fig. 1b,d), similar to results in oocytes^16^.

## Regulation of PIN1 by phosphorylation

PIN polarity and transport activity are regulated by various protein kinases^20–24^. Several phosphorylation sites (phosphosites) have been identified in the hydrophilic loop of long PINs, including three threonine residues (T227, T248 and T286) and four serine residues (S231, S252, S271 and S290) in PIN1^14,22^. We generated non-phosphorylatable and phosphomimetic mutants of the three threonines (T3A and T3E, respectively) or the four serines (S4A and S4D, respectively) and examined their transport activities (Extended Data Fig. 2d). The non-phosphorylatable T3A and S4A mutants had slightly decreased transport activity compared with the wild type when co-expressed with D6PK (Extended Data Fig. 2e). Notably, the efflux activities were not further enhanced in the phosphomimetic mutants when expressed alone (Extended Data Fig. 2e), indicating that the activation of PIN1 by D6PK in HEK293F cells may be owing to phosphorylation at other sites^20^. Mass spectrometry analysis of purified wild-type PIN1 co-expressed with D6PK in HEK293F cells identified 14 phosphosites, including the known ones at S252 and S290 (Extended Data Table 1). Analysis of PIN1 expressed alone also identified ten phosphosites, which may contribute to its weak efflux activity (Extended Data Table 1).

## Structure determination of PIN1

**Extended Data Fig. 3:**
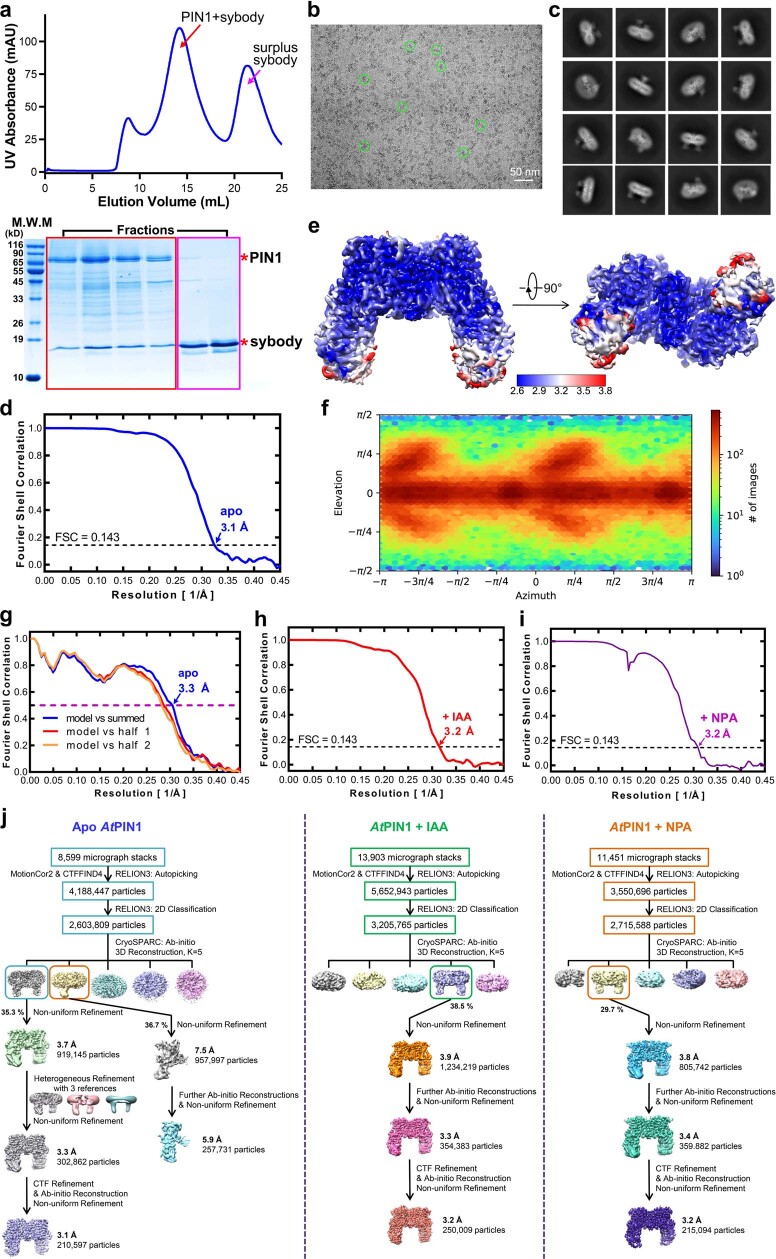
Cryo-EM analysis of PIN1 with Sybody-21. **a**, Representative gel filtration and Coomassie-blue-staining SDS-PAGE results of WT PIN1 bound to Sybody-21. Peaks corresponding to PIN1 plus Sybody-21 and the redundant Sybody-21 are indicated by red and pink arrows, respectively. Independent experiments have been repeated for five times with similar results. **b**, A typical cryo-EM image of the apo PIN1 plus Sybody-21. **c**, Typical 2D classification images of the apo PIN1 plus Sybody-21. **d**, The gold-standard Fourier shell correlation curves for the overall map of the apo PIN1 plus Sybody-21. **e**, The local resolution maps of apo PIN1 plus Sybody-21 sample calculated using ResMap. **f**, Euler angle distribution of the apo PIN1 plus Sybody-21 map. **g**, FSC model curves of the apo PIN1 plus Sybody-21 map. **h**, The gold-standard Fourier shell correlation curves for the overall map of the PIN1 plus IAA and Sybody-21 sample. **i**, The gold-standard Fourier shell correlation curves for the overall map of the PIN1 plus NPA and Sybody-21 sample. **j**, Flowchart for cryo-EM data processing. Full version of all gels and blots are provided in Supplementary Fig. 1.

**Extended Data Fig. 4:**
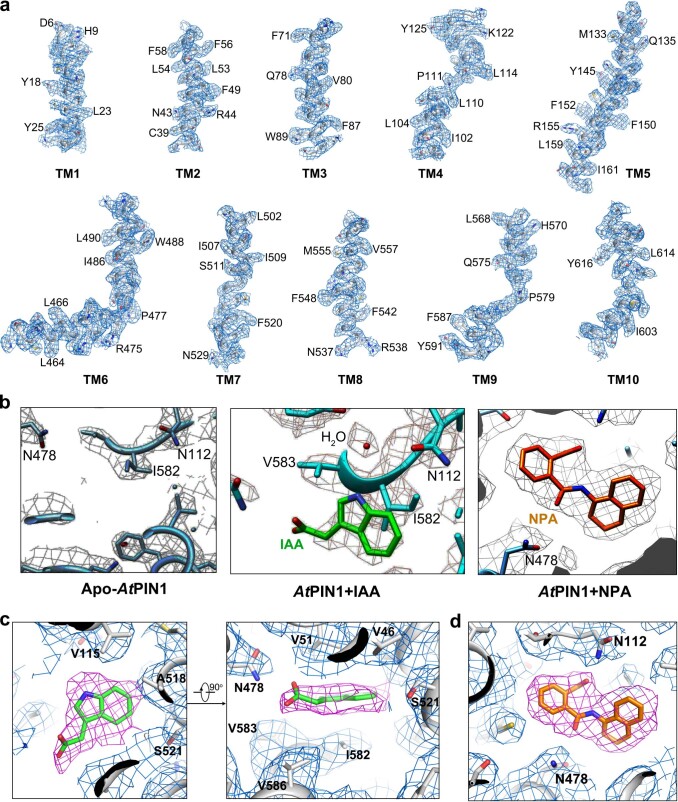
Representative EM densities for the PIN1 maps. **a**, EM densities for the ten transmembrane helices of the apo PIN1 map. Residues with notable side chains are labeled aside. **b**, EM densities for the ligand binding pocket in the apo PIN1 map (left), the IAA-bound map (middle), and the NPA-bound map (right). A clear density is identified in the EM map of the PIN1 plus IAA, or NPA sample, which is docked as an IAA or NPA molecule, respectively. A density modelled as water is also observed between IAA and the N112 residue. **c**, Zoom-in views of the EM density for IAA. **d**, A zoom-in view of the EM density for NPA.

Purified PIN1 protein exhibited good solution behaviour (Extended Data Fig. 2g). To facilitate cryo-electron microscopy (cryo-EM) analysis, we generated synthetic nanobodies (sybodies) using an in vitro nanobody synthesis platform^25^. Using sybodies that could increase the thermal stability of PIN1 in a microscale fluorescent assay^26^, we prepared complex samples and proceeded to structure determination (Extended Data Fig. 3a–c). Using one of the selected sybodies (Sybody-21) (Extended Data Fig. 2h), we determined a dimeric structure of PIN1 (Fig. 1e and Extended Data Fig. 3d,j). The map density was of high quality, enabling de novo model building (Extended Data Figs. 3e–g and  4a). Most of the hydrophilic loop was missing—possibly owing to its intrinsic flexibility—and none of the reported phosphosites were visible, precluding further structural analysis of the regulation of PIN1 by phosphorylation. The sybody bound to a juxtamembrane region of the hydrophilic loop (Fig. 1e). We also identified a monomeric PIN1 during 3D classification (Extended Data Fig. 3j), however, this class did not result in a high-resolution map. Therefore, we focused mainly on analysis of the dimeric PIN1 structure.

## Overall architecture of PIN1

**Extended Data Fig. 5:**
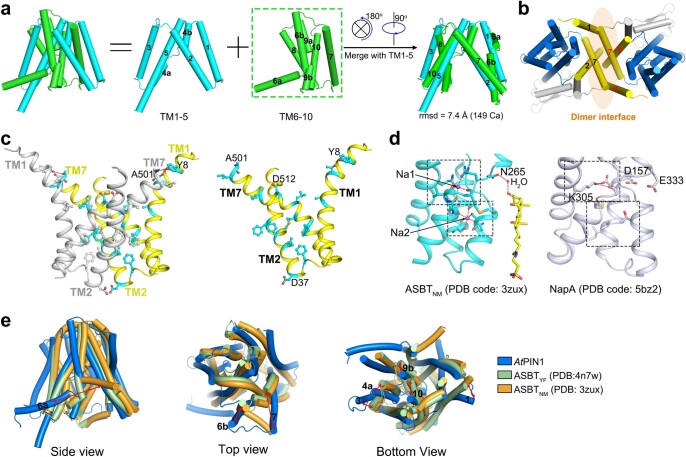
PIN1 shares a NhaA-fold. **a**, The first and last five TMs of PIN1 are structurally inverted repeats. TM6-10 merges well with TM1-5 by two rotational operations as indicated. **b**, The dimer interface of PIN1 is formed by TM1, 2, 7 of each monomer. **c**, The dimer interface mainly consists of hydrophobic residues. **d**, Two sodium binding sites of ASBT_NM_ (left) and the corresponding positions in the Na^+^/H^+^ transporter NapA (right). Left, the sodium ions are shown in purple spheres. N265 and the substrate taurocholate are shown in sticks. The water molecule mediating the interaction between N265 and taurocholate is shown in a red sphere. Right, a lysine residue is present in the Na1 site in NapA and stabilizes the crossover helices through ionic interactions. The Na2 site of NapA is composed of mainly hydrophobic residues as shown in sticks. **e**, Structural alignments between PIN1 and the inward-facing structures of ASBT_YF_ (PDB code: 4N7W) and ASBT_NM_ (PDB code: 3ZUX).

The transmembrane domain of PIN1 has ten transmembrane segments (TM1 to TM10), with both N and C termini located extracellularly as previously reported^10^ (Fig. 1f,g). PIN1 adopts a NhaA fold; this fold was first observed in the bacterial Na^+^/H^+^ antiporter^27^ and is shared by mammalian Na^+^/H^+^ exchangers^28^ and apical sodium-dependent bile acid transporter (ASBT) homologues, such as ASBT_NM_ and ASBT_YF_^32,33^. TM1 to TM5 and TM6 to TM10 form two inverted repeats that can be superimposed well (Extended Data Fig. 5a). TM1, TM2, TM6 and TM7 form the scaffold domain, and TM3 to TM5 and TM8 to TM10 form the transporter domain (Fig. 1g). TM1, TM2 and TM7 constitute the dimer interface, which consists mainly of hydrophobic residues (Extended Data Fig. 5b,c). TM6 is discontinuous, with its first half being tilted, lying almost parallel to the membrane surface (Fig. 1f).

**Fig. 2:**
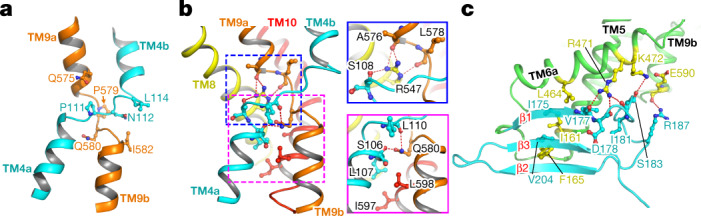
TM4 and TM9 of PIN1 form a crossover structure. **a**, An overview of the crossover structure of TM4 (cyan) and TM9 (orange). **b**, The crossover structure of TM4 and TM9 is stabilized by the positively charged R547 in TM8. Positions corresponding to the two sodium binding sites in ASBT_NM_ are indicated by dashed outlines. Magnified views are shown for each site. **c**, PIN1 contains a cytosolic domain (cyan) composed of three β-strands. Ionic interactions between the transmembrane segments and the β1–β2 linker are shown as red dashed lines.

Two discontinuous, inverted helices—TM4 and TM9—cross over at the centre of the transmembrane domain; such signature helices have been shown to be essential for substrate coordination and transport in ASBTs and Na^+^/H^+^ transporters^28^ (Fig. 2a). Two prolines, P111 of TM4 and P579 of TM9, are located at the crossover, followed by an asparagine (N112) and glutamine (Q580), respectively (Fig. 2a). Notably, a P579L mutation in the *pin1-4* allele was reported to disrupt the function of PIN1, corroborating the essential role of these prolines^29^. In the inward-facing structure of ASBT_NM_, two Na^+^ binding sites were identified, one (Na1) located near the centre of the crossover and the other (Na2) located near the cytoplasm^30^ (Extended Data Fig. 5d). Na^+^/H^+^ transporters have positively charged residues in similar positions, mimicking the effect of sodium ions^31–35^ (Extended Data Fig. 5d). In PIN1, no densities are observed in Na1 or Na2 positions, and the surrounding residue compositions are different. A positively charged residue, R547, is present in the Na1 position and interacts with the backbones of S108, A576 and L578, thus stabilizing TM4 and TM9 in the crossover configuration (Fig. 2b). A neutrally charged residue in the Na2 position, Q580, interacts with the backbones of S106 and L110 (Fig. 2b). PIN1 is also unlikely to bind sodium in the Na2 position, since most of the polar or negatively charged residues that are present in the equivalent site in ASBT_NM_ are replaced by hydrophobic or neutrally charged residues.

A short segment after TM5 (residues 165–211) forms a cytosolic domain comprising three β-strands (Fig. 2c). This domain packs closely with nearby transmembrane segments, including TM5, TM6a and TM9b. The β-strands bind to TM5 and TM6a through mainly hydrophobic interactions, and the β1–β2 linker interacts with TM5 and TM9b through salt bridges. R471 in TM5 interacts with the backbones of V177, D178 and I181 in the β1–β2 loop. K472 in TM5 interacts with the side chain of S183, and E590 in TM9b interacts with R187 (Fig. 2c).

**Fig. 3:**
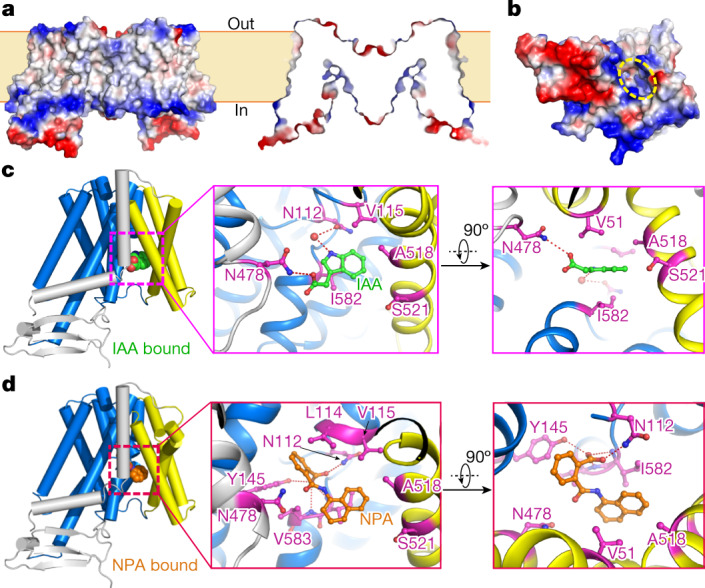
IAA and NPA are coordinated in the intracellular pocket of PIN1. **a**, Side view and section view of the surface electrostatic potential of PIN1 in the apo state. Negative and positive charges are coloured red and blue, respectively. **b**, Overview of the surface electrostatic potential of the PIN1 monomer from the intracellular side. The cavity is indicated with yellow dashed lines. **c**, Coordination of IAA by PIN1. IAA is shown as spheres in the overall view (left). TM1, TM2 and TM7 are coloured yellow; TM6 is coloured grey and transmembrane segments of the transporter domain are coloured marine blue. The IAA molecule and interacting residues are shown as sticks in the magnified views (centre and right). Hydrogen bonds are shown in red dashed lines. **d**, Coordination of NPA by PIN1. NPA is shown in spheres and sticks in the side view (left) and magnified views (centre and right), respectively.

Each PIN1 monomer has a solvent-accessible cavity with a weak positive potential opening to the intracellular side (Fig. 3a,b), suggesting an inward-facing state. Accordingly, PIN1 aligns better to the inward-facing structures of ASBT_NM_ and ASBT_YF_ (Extended Data Fig. 5e), whereas the scaffold domain shows large variations (Extended Data Fig. 5e).

## IAA maps to the intracellular pocket of PIN1

**Extended Data Fig. 6:**
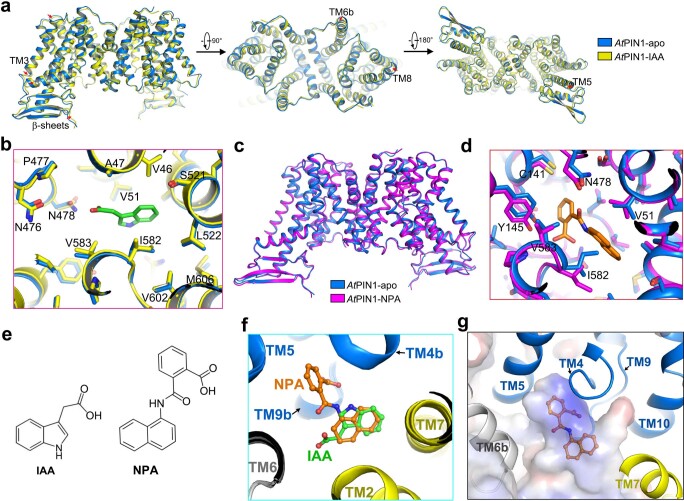
IAA and NPA coordination of PIN1. **a**, Structural comparisons between the apo and IAA-bound structure of PIN1. **b**, Residues surrounding the IAA binding site remain almost identical in the apo and IAA-bound structures. **c**, Structural comparisons between the apo and NPA-bound structure of PIN1. **d**, Residues surrounding the NPA binding site undergo subtle rearrangements. **e**, Chemical structures of IAA and NPA. **f**, Superposition of the IAA-bound and NPA-bound structures of PIN1. **g**, The benzoic group of NPA binds to a pocket formed by TM5 and the crossover regions of TM4 and TM9. Surface electrostatic potential is shown for PIN1.

With the presence of IAA throughout the purification process and before cryo-sample preparations, we obtained an electron microscopy map with an overall resolution of 3.2 Å (Extended Data Fig. 3h,j). An additional electron microscopy density appeared near the bottom of the intracellular cavity, which could be docked with IAA (Extended Data Fig. 4b,c). A small density was also observed between IAA and the crossover region of TM4, which we modelled as a water molecule (Extended Data Fig. 4b). The overall IAA-bound structure was largely the same as the apo state (Extended Data Fig. 6a). Residues around the IAA binding site were nearly identical in both structures (Extended Data Fig. 6b). In the structure, IAA is coordinated through both hydrogen bonding and hydrophobic interactions (Fig. 3c). The carboxyl group of IAA interacts with N478 in TM6b. The water molecule bridges the interaction between the amide group of IAA imidazole ring and N112. The imidazole ring is packed between V51 and I582, both of which are located within 4 Å of the ring plane (Fig. 3c). All interacting residues (V51, N112, N478 and I582) are highly conserved in PINs (Extended Data Fig. 1). N112, N478 and I582 are invariant, whereas V51 is substituted by a leucine and isoleucine in PIN5 and PIN8, respectively.

## NPA maps to the intracellular pocket of PIN1

We also determined the NPA-bound structure of PIN1 (Extended Data Fig. 3i,j). In the bottom of the intracellular pocket, we identified a density that fitted perfectly with NPA, which is in a similar position to the IAA binding site (Fig. 3d and Extended Data Figs. 4b,d and  6f). The NPA-bound structure is largely the same as the apo structure, although residues around NPA are slightly altered, providing better coordination (Extended Data Fig. 6c,d). NPA is tightly coordinated (Fig. 3d): the naphthalene group is packed between V51 and I582, the carboxyl group forms hydrogen bonds with the side chains of N112 and Y145 and the backbone atoms of I582 and V583, and the benzene ring is stacked among Y145, L114 and V583. Thus NPA preserves the binding mode between IAA and PIN1, since V51, N112 and I582 participate in binding of both ligands. Indeed, NPA and IAA share chemical similarity, containing both a hydrophobic ring and a negatively charged group (Extended Data Fig. 6e). However, the interaction between NPA and PIN1 includes more hydrogen bonds owing to the additional benzoic acid group in NPA. Notably, this group protrudes into the pocket formed by TM5 and the crossover region (Extended Data Fig. 6g). For NhaA fold transporters, the crossover region is thought to undergo local rearrangements, leading to conformational changes that fulfill the transport cycle^28^. The benzoic acid group of NPA may reduce the mobility of the crossover region, and thus arrest PIN1 in the inward-facing conformation.

## IAA and NPA binding to PIN1

**Fig. 4:**
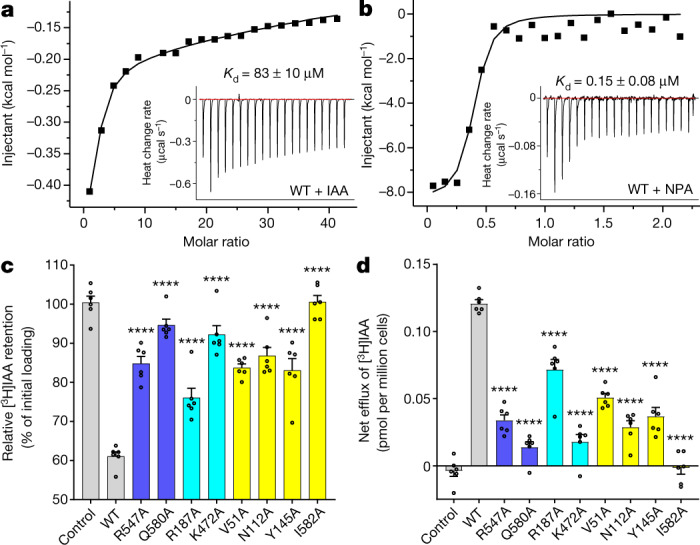
NPA competes with IAA and binds to a similar position in PIN1. **a**, ITC results for IAA binding to wild-type PIN1 at pH 7.0. Inset graphs show the original heat-change recordings. **b**, ITC results for NPA binding to wild-type PIN1 at pH 7.0. **c**, Characterization of auxin transport for wild-type (WT) and mutant PIN1 in HEK293F cells using the auxin efflux assay. **d**, Net IAA efflux with wild-type and mutant PIN1. Six independent experiments were performed for each construct in **c**,**d**. One-way ANOVA with Dunnett’s multiple comparisons test. ****P < 0.0001 for mutants versus wild type. Data are mean ± s.e.m. Source data.

**Extended Data Fig. 7:**
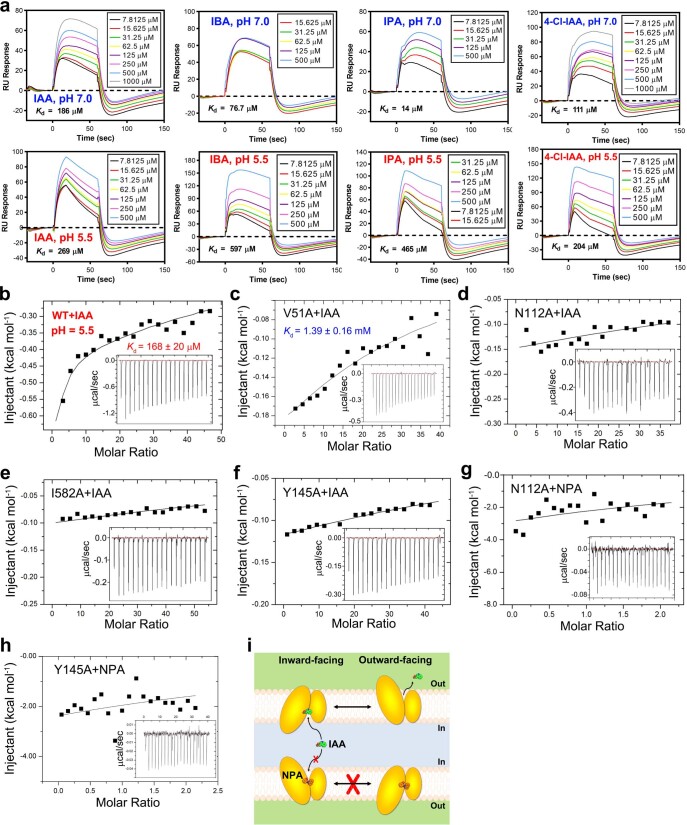
Binding between PIN1 and IAA, NPA or other natural auxins. **a**, The binding affinity between PIN1 and IBA, IPA, or 4-Cl-IAA under pH 7.0 or 5.5 as examined by SPR, respectively. Under acidic pH, the binding affinities decreased dramatically for IBA and IPA, while for 4-Cl-IAA, the *K*_d_ had an about two-fold increase. These results suggest that PINs can bind range of natural auxins but whether PIN1 can transport these auxins awaits further studies. **b**, IAA-ITC results for the WT PIN1 at pH 5.5. **c–f**, IAA-ITC results for the PIN1 V51A, N112A, I582A and Y145A mutants at pH 7.0, respectively. No apparent binding are derived for the N112A, I582A and Y145A mutants. For V51A, the fitted binding affinity is 1.39 ± 0.16 mM, 17-fold higher than that of the WT PIN1. **g**, **h**, NPA-ITC results for the PIN1 N112A and Y145A mutants. **i**, Cartoon model for the alternating access mechanism of IAA transport by PIN1 and inhibition by NPA.

We first tested the binding affinity between IAA and PIN1 at physiological cytosolic pH (pH 7.0). We determined the dissociation constant (*K*_d_) by isothermal titration calorimetry (ITC) (83 μM) and surface plasmon resonance (SPR) (186 μM) (Fig. 4a and Extended Data Fig. 7a). At pH 5.5, the *K*_d_ values were approximately 168 μM and 268 μM by ITC and SPR, respectively (Extended Data Fig. 7a,b), both within a similar range and showing an increase of about twofold only at pH 5.5 relative to pH 7.0, indicating that the binding between PIN1 and IAA is not highly affected by pH. PIN1 can bind also other natural auxins, including indole-3-butyric acid, indole-3-propionic acid and 4-chloroindole-3-acetic acid (Extended Data Fig. 7a).

To confirm the location of the substrate binding site in the PIN1 structure, we generated several variants and examined IAA binding using ITC (Extended Data Fig. 7c–e). Mutation of N112 and I582 to alanine prevented IAA binding (Extended Data Fig. 7d,e). The *K*_d_ for the V51A mutant was 1.39 mM, about 17-fold higher than for the wild type, indicating a strongly reduced binding affinity (Extended Data Fig. 7c). The N478A mutation had detrimental effects on expression level and solution behaviour in gel filtration, precluding further analysis, indicating a function for N478 in structural stabilization.

We also examined the binding between NPA and wild-type PIN1. Compared with IAA, NPA had a markedly higher affinity, with a *K*_d_ value of about 0.15 μM (Fig. 4b), consistent with previous indications that NPA is an effective inhibitor and shows high-affinity binding to the integral components of plant membranes^36^. Binding between NPA and the N112A mutant is largely impaired, consistent with the structural observations that N112 is involved in both IAA and NPA recognition (Extended Data Fig. 7g). No binding was observed between NPA and the Y145A mutant (Extended Data Fig. 7h). Notably, the binding between IAA and Y145A is also impaired (Extended Data Fig. 7f). Whether Y145 is involved in direct IAA binding in other states during transport or has a structural role in maintaining the critical binding site requires further investigation.

These binding assays supported the auxin binding site predicted by the structural studies and confirmed that NPA binds with high affinity at the same site.

## IAA efflux activities of *pin1* mutants

Our structural analyses identified residues that may be essential for the stability and transport activity of PIN1, including R547 and Q580 in similar locations to the Na^+^ binding sites in ASBT_NM_, charged residues (R187, R471 and K472) mediating the interactions between the cytosolic β-sheet domain and transmembrane segments, and the IAA-coordinating residues V51, N112, N478 and I582. We generated alanine mutants of these residues and measured their auxin efflux activities. R547A and Q580A mutants showed markedly increased [^3^H]IAA retention and decreased net efflux, indicating largely impaired transport activities (Fig. 4c,d and Extended Data Fig. 2a). This suggests that these residues are essential for the transport activity of PIN1, possibly by mimicking the role of Na^+^ in ASBT_NM_ and stabilizing the crossover helices.

Next, we analysed residues mediating the interactions between the cytosolic β-sheet and transmembrane segments. Mutating R471 to alanine led to barely detectable expression (Extended Data Fig. 2a). The R187A mutant exhibited a slightly decreased efflux activity, whereas K472A showed a considerable impairment (Fig. 4c,d). Together, these results suggest that the structural integrity of this domain is essential for the transport activity. Notably, the sequence of this segment is conserved only among long PINs (Extended Data Fig. 1). Its detailed functions await further study.

We also characterized the transport activities of the IAA-coordinating residues using V51A, N112A and I582A mutants, but not N478A, which exhibited impaired protein expression. V51A, N112A and I582A mutations resulted in reduced efflux activities, especially I582A (Fig. 4c,d). Consistent with the ITC result, the IAA transport activity of the Y145A mutant was largely impaired (Fig. 4c,d), suggesting its involvement in both NPA inhibition and IAA efflux. Together with the results from ITC, the observed binding pocket represents an essential IAA-coordinating site in PIN1. Specifically, N112 of PIN1 is conserved in ASBTs, whereas in the Na^+^/H^+^ transporters, it is replaced by a negatively charged aspartic or glutamic acid residue (Extended Data Fig. 5d). This reinforces the critical role of the crossover helices in substrate recognition and provides strong functional support for our PIN1 structure.

## Discussion

Here we have characterized the architecture and substrate binding profile of PIN1, the main PIN auxin exporter in *Arabidopsis*. An elevator-like alternating access mechanism has been proposed for the characterized NhaA fold transporters, on the basis of available outward- and inward-facing structures and the accompanying functional analyses^37,38^. In this view, the transporter domain picks up the substrate on one side of the membrane, slides along the scaffold domain to undergo conformational changes, and releases the substrate to the other side of the membrane (Extended Data Fig. 7i). Although a similar model is plausible for PIN1-based IAA transport, confirmation of the mechanism requires the capture of more states during the transport cycle, in particular, an outward-facing structure. NPA and IAA directly compete for the intracellular binding pocket of PIN1. Owing to the higher affinity between NPA and PIN1, IAA is prohibited access to the binding pocket once NPA is bound, even at higher concentrations (Extended Data Fig. 7i).

The source of the energy used for PIN1-mediated auxin transport remains unknown. Owing to the chemical nature of IAA as a weak acid, the proportions of the protonated or deprotonated form vary at different pH values. In the chemiosmotic hypothesis of auxin transport, the hydrophobic IAAH protonated in the apoplast diffuses easily through the lipid bilayer. Once in the neutral or basic intracellular pH environment, it exists dominantly in the deprotonated IAA^−^ form and its export facilitated by transporters such as PINs is essential^39–41^. The results of our in vivo assay support this model, showing that without PIN1 expression, almost no IAA efflux occurs (Fig. 1c,d). The same pH gradient at the plasma membrane has been assumed to provide the energy for auxin export. However, we did not detect any significant difference in the relative IAA retention between pH 5.5 and 6.5 (Extended Data Fig. 2f). Similarly, we did not detect much influence of pH on IAA binding to PIN1 (Extended Data Fig. 7a,b). Both findings indicate that PIN transport may not utilize the pH gradient. Nonetheless, this requires further validation, preferably using an in vitro transport system.

Together, our studies provide structural insights into auxin efflux and the mechanism of polar auxin transport. They set up a framework for future structure-based functional analysis of PINs and the design of auxin analogues for agricultural use.

## Methods

### Protein expression and purification

The DNA sequence of full-length *A. thaliana* PIN1 is publicly available at Uniprot (https://www.uniprot.org) with an accession code of Q9C6B8, and was cloned into the pCAG vector (Invitrogen) with the N-terminal Flag tag for cryo-EM sample preparation or an extra C-terminal AVI tag for sybody selection. All PIN1 variants were generated with the N-terminal Flag tag in the same way. The HEK293F cells (Sino Biological Inc.) were transfected with plasmids of PIN1 when reaching a density of 3 × 10^6^ cells per ml. The HEK293F cell line used has not been authenticated and has been tested negative for mycoplasma contamination. For 1-l cell culture, 1.5 mg plasmids were premixed with 4 mg linear polyethylenimines (PEI) (Polysciences) in 50 ml medium for 30 min. The mixture was then added into cell culture followed by a 15-min incubation. Transfected cells were cultured at 37 °C for 60 h before collected by centrifugation at 3,000 rpm for 10 min. Pellets were resuspended in the lysis buffer containing 25 mM HEPES, pH 7.4, 150 mM NaCl, 1.5% (w/v) DDM (Anatrace) and the protease inhibitor cocktail containing 1 mM phenylmethylsulfonyl fluoride, aprotinin (1.3 mg ml^−1^), pepstatin 3 (0.7 mg ml^−1^) and leupeptin (5 mg ml^−1^). After incubation at 4 °C for 2 h, supernatant was isolated by centrifugation at 14,000 rpm for 1 h and incubated with anti-Flag M2 affinity gel (Sigma) at 4 °C for 45 min. The resin was rinsed 3 times with 10 ml buffer each time containing 25 mM HEPES, pH 7.4, 150 mM NaCl, 0.06% (w/v) glyco-diosgenin (GDN) (Anatrace). Protein was then eluted with the wash buffer plus 200 μg ml^−1^ Flag peptides. Eluent was concentrated with a 100-kDa cut-off Centricon (Millipore) and further purified by size-exclusion chromatography using a Superose 6 Increase column (GE Healthcare) in buffer containing 25 mM HEPES, pH 7.4, 150 mM NaCl and 0.02% (w/v) GDN. For the determination of the IAA-bound structure, 10 mM IAA sodium salt (Sigma) was added during the whole process starting from protein extraction by detergent. The gel filtration buffer contains 10 mM IAA sodium salt, 25 mM HEPES, pH 7.4, 150 mM NaCl and 0.02% (w/v) GDN.

### Cell-based auxin transport assays

*A. thaliana* D6PK (Uniprot accession code: Q9FG74), the wild type or mutants of PIN1 were subcloned into the pCAG vector, respectively. HEK293F cells at a density of 1.5 × 10^6^ cells per ml were transfected with the empty vector or PIN1 constructs, or co-transfected with PIN1 and D6PK at a mass ration of 3:1. Twenty-four hours after transfection, cells were collected by centrifugation and resuspended for the loading assay or efflux assay. For all the characterizations of PIN1 mutants, cells were co-transfected with D6PK unless otherwise noted. Cell counts were determined using Coulter counting and microscopic visualization. For all assay system, a 500 μl aliquot of cell suspension contains 3 × 10^6^ cells. For the [^3^H]IAA accumulation assay, cells were resuspended and incubated at 37 °C with the PBS citrate buffer, pH 5.5 (10 mM Na_2_HPO_4_, 1.8 mM KH_2_PO_4_, 2.7 mM KCl, 137 mM NaCl, pH adjusted by citric acid anhydrous) containing 16 nM [^3^H]IAA (specific activity 25 Ci mmol^−1^, American Radiolabeled Chemicals). The loading process was stopped by centrifugation at indicated time points. Cells were then washed twice with the ice-cold PBS buffer, pH 7.4, and resuspended with the same buffer plus 1% Triton X-100 for cell lysis. The radioactivity in the cell lysis was counted using liquid scintillation counting (Tri-Carb 2910TR, PerkinElmer). For the auxin efflux assay, cells were first loaded in PBS citrate buffer, pH 5.5, plus 40 nM [^3^H]IAA for 5 min, then washed and resuspended with [^3^H]IAA-free PBS citrate buffer. 500 μl aliquots were taken immediately after resuspension (defined as the zero time point) or at other indicated time points. Cells were centrifuged and washed twice with 1 ml ice-cold PBS buffer, pH 7.4, and resuspended with the same buffer plus 1% Triton X-100 for scintillation counting. For the PIN1 mutants, [^3^H]IAA retention is presented as residual radioactivity measured at 10 min relative to 0 min, and net efflux is expressed as the amount of radiolabeled [^3^H]IAA measured at 0 min minus the retained [^3^H]IAA measured at 10 min. For NPA inhibition assays, cells were incubated with [^3^H]IAA in the presence of 10 μM NPA (Sigma).

### Biotinylation of PIN1 for sybody selection

Purified PIN1 (1.3 mg ml^−1^) with a N-terminal Flag tag and an extra C-terminal AVI tag was biotinylated using BirA (66 μg ml^−1^) in a buffer containing 150 mM NaCl, 0.02% (w/v) GDN, 10 mM magnesium acetate, 5 mM ATP, 28 μM biotin and 25 mM HEPES pH 7.4 at 4 °C. After 16 h of reaction, the biotinylation was continued by adding 1 mM of ATP and 13 μg ml^−1^ of BirA for another 5 h. The reaction mix was incubated with Ni-NTA resin to remove His-tagged BirA. The flow-through fractions were fractioned by gel filtration on a Superose 6 Increase 10/300 GL column (GE Healthcare). Fractions containing PIN1 were concentrated and flash-frozen in liquid nitrogen in small aliquots and stored at −80 °C before use.

### In vitro translation and selection of sybodies

For in vitro translation of sybodies, a 9.3-μl mix (PUREfrex 2.1 kit, Genefrontier) containing 1.8 μl of nuclease-free water, 4 μl of solution I, 0.5 μl of solution II, 1 μl of solution III, 0.5 μl of 10 mM cysteine, 0.5 μl of 80 mM reduced glutathione, 0.5 μl of 60 mM oxidized glutathione, and 0.5 μl of 1.875 mg ml^−1^ disulfide isomerase DsbC (Genefrontier) was first incubated at 37 °C for 5 min in a PCR cycler. The reaction was initiated by adding 0.7 μl of sybody mRNA library. After 30 min of incubation at 37 °C, the mix was added with 100 μl of ice-old panning solution (0.02% GDN, 150 mM NaCl, 50 mM magnesium acetate, 0.5% (w/v) BSA, 0.5% (w/v) heparin, 1 μl RnaseIn, 50 mM Tris-acetate pH 7.4) and centrifuged at 20,000*g* for 5 min at 4 °C. The supernatant was mixed with 50 nM of biotinylated PIN1 and incubated on ice for 20 min for solution panning. The mixture was added into 12 μl of Streptavidin beads (Dynabeads Myone Streptavidin T1) pre-equilibrated with WTB buffer (50 mM Tris-acetate pH 7.4, 150 mM NaCl, 50 mM magnesium acetate) supplemented with 0.5% BSA and incubated for 10 min. The beads were washed thrice with WTB-D buffer (0.008% GDN in WTB buffer) before being eluted with RD elution buffer (100 μg ml^−1^ yeast RNA, 150 mM NaCl, 50 mM EDTA, 50 mM Tris-acetate pH 7.4). The eluted mRNA was purified using Rneasy Kit (Qiagen) and reverse-transcribed using the primer (5′-CTTCAGTTGCCGCTTTCTTTCTTG-3′) and 2 μl of RNA transcriptase (Agilent) in a 40-μl reaction. cDNA was purified (Macherey-Nagal) and PCR-amplified using the primer pairs as follows: (5′-ATATGCTCTTCTAGTCAGGTTCAGCTGGTTGAGAGCG-3′ and 5′-TATAGCTCTTCATGCGCTCACAGTCACTTGGGTACC-3′ for the Concave and Loop libraries, and 5′-ATATGCTCTTCTAGTCAAGTCCAGCTGGTGGAATCG-3′ and 5′-TATAGCTCTTCATGCAGAAACGGTAACTTGGGTGCCC-3′ for the Convex library. Amplified DNA products were purified and digested using the Type IIs restriction enzyme BspQI, and ligated into the vector pDX_init pre-treated with the same enzyme. The ligation products were transformed into *Escherichia coli* SS320 competent cells by electroporation to generate libraries for phage display.

Three rounds of phage display with alternating beads under increasingly stringent conditions were performed. The first round was performed in a 96-well plated coated with 67 nM neutravidin (Thermo Fisher Scientific). Purified phage was incubated with 50 nM of biotinylated PIN1 for 20 min at room temperature (20–22 °C) before being added into the 96-well plate. After three times of washing using TBS-D buffer (0.01% GDN, 150 mM NaCl, 20 mM Tris-HCl pH 7.4), phage particles were eluted using 0.25 mg ml^−1^ of trypsin. Eluted phage particles were amplified and applied to the second round of phage display. To this end, 12 μl of MyOne Streptavidin C1 beads were incubated with 50 nM biotinylated PIN1 and phage particles in 100 μl of panning solution for 10 min. Weaker binders were challenged by incubating the beads with 5 μM of non-biotinylated PIN1 for 3 min at room temperature. The phage display process was repeated in the same manner as the second round except that the concentration of the biotinylated and non-biotinylated PIN1 was reduced by a factor of 10. Sybody-encoding fragments in the phagemid were exchanged into the pSb_init vector using fragment-exchange (FX) cloning for further enzyme-linked immunosorbent assay^42^.

### Enzyme-linked immunosorbent assay

The pSb_init plasmids obtained above were transformed into *E. coli* MC1061 competent cells. Single colonies were inoculated and grown at 37 °C for 4 h in a 96-well plate. The pre-culture was then seeded into 1 ml of Terrific broth (1.2% tryptone, 2.4% yeast extract, 1% NaCl, 4 ml glycerol, 0.231% KH_2_PO_4_, 1.254% K_2_HPO_4_) supplemented with 25 μg ml^−1^ chloramphenicol and cultured at 37 °C. After 2 h, the temperature was dropped to 22 °C. After another 1.5 h, sybody expression was induced with 0.02% (w/v) L-(+)-arabinose for 17 h. Cell pellets were collected and lysed with TES buffer (0.5 μg ml^−1^ lysozyme, 0.5 mM EDTA, 20% (w/v) sucrose, 50 mM Tris-HCl pH 8.0). After further incubation with TBS buffer (150 mM NaCl, 20 mM Tris-HCl pH 7.4) supplemented with 1 mM MgCl_2_ for 30 min, the mixture was centrifuged at 3,220 g for 30 min at 4 °C. The supernatant fractions containing sybodies were used for ELISA.

A Maxi-Sorp plate (Thermo Fisher Scientific) was incubated with protein A in PBS at 4 °C for 16 h. Excess protein A was removed and the plate was washed once using TBS buffer, blocked with TBS supplemented with 0.5% BSA, and washed thrice with TBS. Anti-Myc antibody (Sigma) at 1:2,000 dilution in TBS-BSA-D buffer (TBS supplemented with 0.5% (w/v) BSA and 0.02% GDN) was added to the 96-well plate to bind with protein A. Excess antibodies were then washed three times using the TBS-D buffer (TBS supplemented with 0.01% GDN). Ten microliters of periplasmic extract as mentioned above were added into the 96-well plate for 20 min incubation at room temperature. The plate was then washed three times using TBS-D buffer. 50 nanomolar biotinylated PIN1 or a control protein was added to the plate. After incubation at room temperature for 20 min, the plate was washed three times with TBS-D buffer. Streptavidin conjugated with horseradish peroxidase (HRP) was added to each well and the plate was incubated at room temperature for 20 min. The plate was washed thrice using the TBS-D buffer before being developed with the ELISA developing buffer (51 mM Na_2_HPO_4_, 24 mM citric acid, 0.006% (v/v) H_2_O_2_, 0.1 mg ml^−1^ 3,3′,5,5′-tetramethylbenzidine). ELISA signal was detected by absorbance at 650 nm in a plate reader.

### Bio-layer interferometry assay

The binding affinity of PIN1 and sybody was measured by BLI with an Octet RED96 system (ForteBio) using a HIS1K biosensor at 30 °C. The sensor was first equilibrated in kinetic buffer (25 mM HEPES pH 7.4, 150 mM NaCl, 0.01% GDN) for 10 min. For BLI measurement, the baseline phase was recorded for 120 s. The loading phase was monitored by soaking the sensor in 2 μg ml^−1^ His-tagged sybody in the kinetic buffer. The binding was monitored by soaking the sensor in various concentrations of the PIN1 analyte for 360 s, and the dissociation phase was recorded by bathing the sensor in the PIN1-free buffer for 600 s. Data were fitted for a 1:1 stoichiometry for the equilibrium dissociation constant *K*_D_, association rate constant *K*_on_, and dissociation rate constant *K*_off_ calculations using Octet Data Analysis software version 9.0.

### Microscale fluorescent thermal stability assay

*N*-(4-(7-diethylamino-4-methyl-3-coumarinyl) phenyl) maleimide (CPM, Sigma) assay was used to test the thermal stability of PIN1 with different sybodies. CPM dye was dissolved in dimethyl sulfoxide (DMSO) at 4 mg ml^−1^ and diluted 40-fold into a buffer containing 25 mM HEPES pH 7.4, 150 mM NaCl, 0.025% DDM prior to use. Two micrograms of purified PIN1 protein was mixed with the sybody (1:4 molar ratio) and incubated on ice for 30 min. Diluted CPM (1.5 μl) was added to the protein and mixed thoroughly on ice in darkness. The reaction mixture (20 µl in total) was heated in a controlled manner with a ramp rate of 3 °C min^−1^ in a LightCycler480 (Roche) real-time PCR instrument. The excitation wavelength was set at 440 nm, and the emission wavelength was set at 488 nm and the fluorescence intensity was continuously measured. Assays were performed over a temperature range starting from room temperature and ending at 90 °C.

### Cryo-EM sample preparation and data acquisition

To prepare PIN1 and the sybody (sybody-21 used for final structure determination) complex, purified PIN1 and sybody-21 were mixed together with a molar ratio of ~1:3 and then injected to gel filtration. No dilution was performed for the purified sybody. For the apo or IAA-bound PIN1 cryo-EM sample preparation, aliquots (4 µl) of the purified protein was added to the glow discharged holey carbon grids (Quantifoil Au R1.2/1.3, 300 mesh), blotted with a Vitrobot Mark IV (Thermo Fisher Scientific) using 4 s blotting time with 100% humidity at 8 °C, and plunged into liquid ethane cooled by liquid nitrogen. For the NPA-bound PIN1 cryo-EM sample preparation, purified PIN1 was first incubated with NPA (Sigma) at a final concentration of 100 μM at room temperature for 30 min, and then applied to the grid. The grid was loaded into a Titan Krios (FEI) electron microscope operating at 300 kV, equipped with the BioQuantum energy filter and a K2 or K3 direct electron detector (Gatan). Images were recorded using EPU or SerialEM in the super-resolution mode. Defocus values varied from −1.5 to −2.3 μm. Image stacks were acquired with an exposure time of 3 s and dose-fractionated to 32 frames with a total dose of 50 e^−^ Å^−2^.

### Image processing

A flowchart for the data processing is presented in Extended Data Fig. 3j. Motion correction and dose weighting were performed using the RELION 3.1 implementation of MotionCor2^43,44^. Defocus values were estimated by CTFFIND4^45^. After manual checking, good micrographs were selected for automatically particle picking using RELION. Picked particles were extracted and applied to 2D classification. Then particles from good classes were imported into cryoSPARC (v.3.2.0)^46^ for further data processing. For the apo structure analysis, particles were classified with an ab initio 3D reconstruction into five classes. Two classes representing the dimeric and monomeric PIN1, respectively, were selected for non-uniform refinement. After non-uniform refinement, an EM map at 3.7 Å was obtained for the dimeric form with 919,145 particles. Heterogeneous refinement with three manually generated low-pass filtered references and a subsequent non-uniform refinement resulted in a map with an overall resolution of 3.3 Å with 302,862 particles. Further contrast transfer function refinement, ab initio classifications and non-uniform refinement using cryoSPARC further improved the resolution. Finally, a map with an overall resolution of 3.1 Å from 210,597 particles was achieved for the apo, dimeric state of PIN1, as estimated with the gold-standard Fourier shell correlation at a 0.143 criterion with a high-resolution noise substitution method^47,48^. Local resolution variations were estimated using ResMap^49^. For the monomeric state, a final EM map with an overall resolution of 5.9 Å from 257,731 particles was achieved. For the data processing of the IAA-bound, dimeric state, similar procedures were carried out and an EM map with an overall resolution of 3.2 Å using 250,009 particles was obtained. No class corresponding to the monomeric state of PIN1 was observed during ab initio 3D reconstruction. For the data processing of the NPA-bound, dimeric state, similar procedures were carried out and an EM map with an overall resolution of 3.2 Å using 215,094 particles was obtained.

### Model building and refinement

The 3.1 Å map for the apo, dimeric PIN1 was used for de novo model building by COOT^50^. Bulky residues such as Phe, Tyr, Trp and Arg were used to guide the sequence assignment, and the chemical properties of amino acids were considered to facilitate model building. Structure refinements were carried out by PHENIX in real space^51^. Overfitting of the model was monitored by refining the model in one of the two independent maps from the gold-standard refinement approach and testing the refined model against the other map. The refined model was docked into the map for the IAA, or NPA-bound, dimeric PIN1, respectively, and further refined by PHENIX. Statistics of the 3D reconstruction and model refinement can be found in Extended Data Table 2.

### Isothermal titration calorimetry

The binding affinities between IAA and PIN1 variants were measured with a MicroCal iTC200 microcalorimeter (MicroCal). Purified wild-type or mutant PIN1 in the buffer containing 0.02% GDN, 25 mM HEPES pH 7.0 (or MES pH 5.5) and 150 mM NaCl were concentrated to about 0.05 mM for isothermal titration calorimetry titration. The protein was titrated by 5 mM IAA dissolved in an identical buffer to that used for size-exclusion chromatography at 25 °C. To measure the binding affinities between NPA and PIN1, 100 μM NPA in the buffer containing 1% DMSO, 0.02% GDN, 25 mM HEPES pH 7.0 and 150 mM NaCl was used to titrate to 0.01 mM purified protein in the identical buffer. Data were fitted using the software Origin 7.0 (MicroCal).

### Surface plasmon resonance

SPR experiments for IAA, indole-3-butyric acid (IBA), indole-3-propionic acid (IPA) and 4-chloroindole-3-acetic acid (4-Cl-IAA) were carried out on a Biacore 8K system (Cytiva) at 25 °C with a flow rate of 30 μl min^−1^. Purified wild-type PIN1 protein were immobilized onto the series S CM5 sensor chips (Cytiva) by amine-coupling chemistry. Ligands at different concentrations were flowed over the chip surface in the pH 7.0 buffer (25 mM HEPES, pH 7.0, 150 mM NaCl, 0.01% GDN, 2% DMSO) or pH 5.5 buffer (25 mM MES, pH 5.5, 150 mM NaCl, 0.01% GDN, 2% DMSO). Data were analysed with the Biacore Insight Evaluation Software Version 3.0.12 using steady state affinity binding model.

### Protein sequences and coordinates

Sequences for the eight PINs and D6PK in *A. thaliana* are publicly available at Uniprot (https://www.uniprot.org) with the following accession codes: PIN1: Q9C6B8, PIN2: Q9LU77, PIN3: Q9S7Z8, PIN4: Q8RWZ6, PIN5: Q9FFD0, PIN6: Q9SQH6, PIN7: Q940Y5, PIN8: Q9LFP6 and D6PK: Q9FG74. Coordinates for the ASBTs and NapA are publicly available at the Protein Data Bank (PDB) with the following accession codes: ASBT_NM_: 3ZUX, ASBT_YF_: 4N7W and NapA: 5BZ2.

### Reporting summary

Further information on research design is available in the Nature Research Reporting Summary linked to this article.

## Online content

Any methods, additional references, Nature Research reporting summaries, source data, extended data, supplementary information, acknowledgements, peer review information; details of author contributions and competing interests; and statements of data and code availability are available at 10.1038/s41586-022-05143-9.

### Supplementary information

Supplementary Figure 1 Uncropped western blot or Coomassie-blue staining of SDS–PAGE gel scans.

Reporting Summary

### Source data

Source Data Fig. 1

Source Data Fig. 4

Source Data Extended Data Fig. 2

## Figures and Tables

**Extended Data Table 1:**
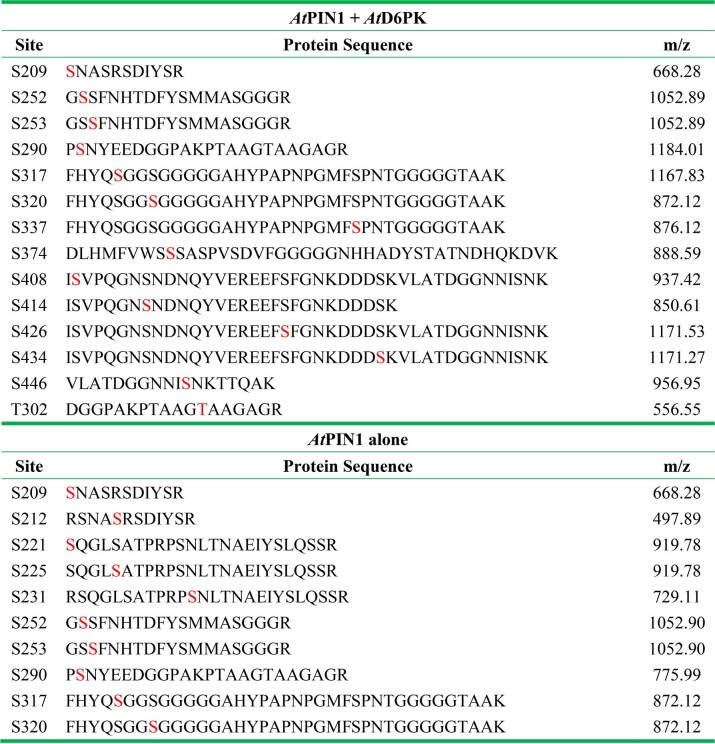
Mass spectrometry analysis of phosphorylation sites of purified PIN1 protein co-expressed with D6PK, or expressed alone in HEK293F cells

Residues identified to be phosphorylated are shown in red.

**Extended Data Table 2:**
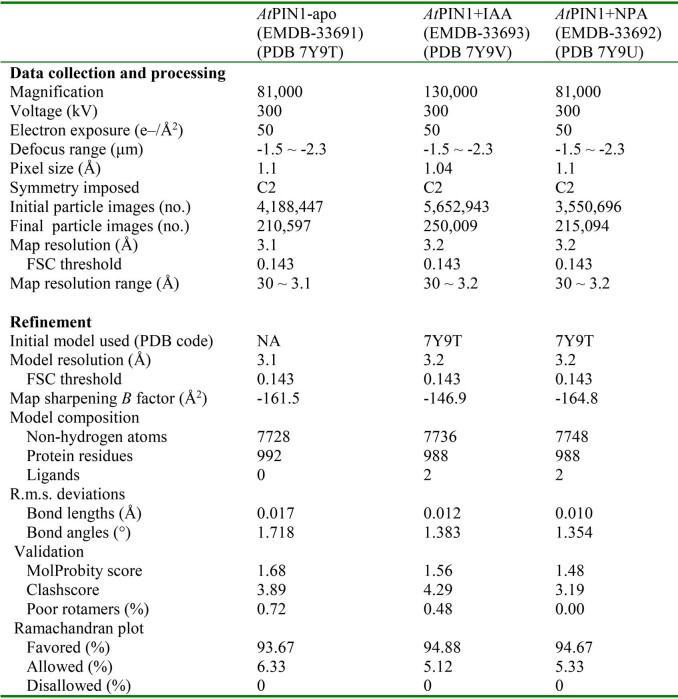
Statistics of cryo-EM data collection, processing, model refinement and validation

## Data Availability

The 3D cryo-EM density maps of the apo, IAA-bound and NPA-bound dimeric-state *A. thaliana* PIN1 have been deposited in the Electron Microscopy Data Bank under the accession numbers EMD-33691, EMD-33693 and EMD-33692, respectively. Coordinates for the apo, IAA-bound and NPA-bound structure models have been deposited in the Protein Data Bank (PDB) under the accession codes 7Y9T, 7Y9V and 7Y9U, respectively. Source data are provided with this paper.
