# Supplementary material for: Structural insights into auxin recognition and efflux by Arabidopsis PIN1
Source: Nature. 2022 Aug 2;609(7927):611–5. doi: 10.1038/s41586-022-05143-9 (PMC9477737; doi:10.1038/s41586-022-05143-9)
Supplement: Supplementary file 1 — Uncropped western blot or Coomassie-blue staining of SDS–PAGE gel scans. [file 41586_2022_5143_MOESM1_ESM.pdf]

nature portfolio

https://doi.org/10.1038/s41586-022-05143-9

Supplementary information

Structural insights into auxin recognition and efflux by *Arabidopsis* PIN1

In the format provided by the authors and unedited

Extended Data Fig. 2a

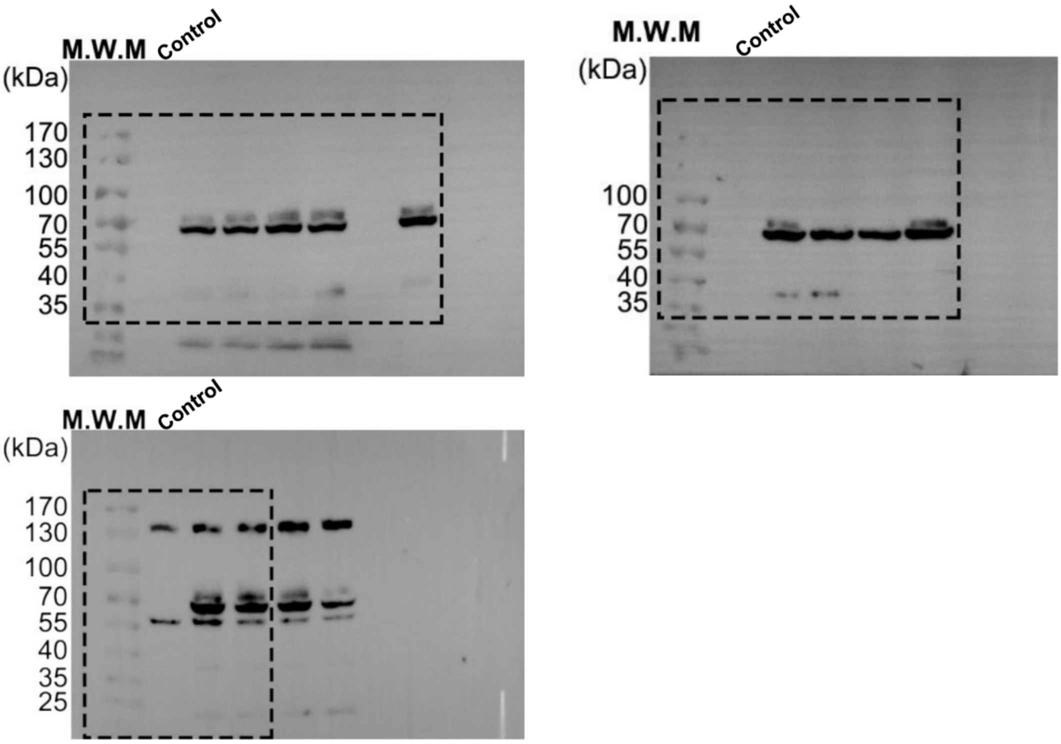

Extended Data Fig. 2d

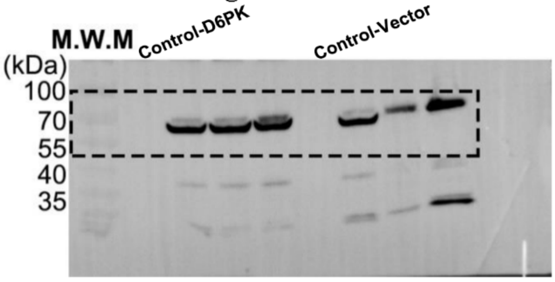

Extended Data Fig. 2g

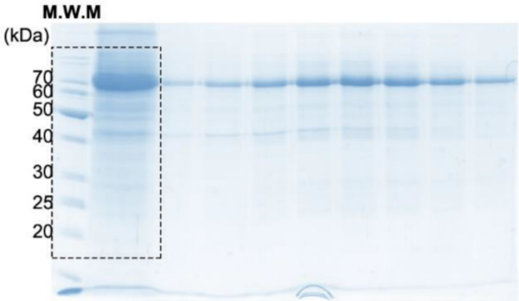

Extended Data Fig. 3a

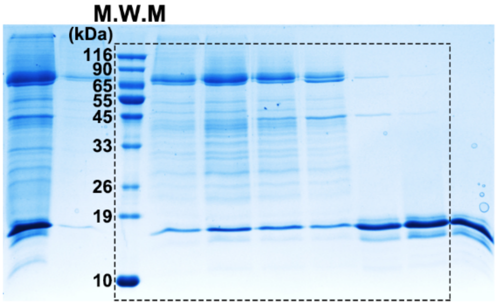

Supplementary Figure 1. Uncropped western blot or Coomassie-blue staining SDS-PAGE gel scans. The cropped areas for use in the figures are indicated by a black dashed box.
