# Supplementary material for: Structural insights into auxin recognition and efflux by Arabidopsis PIN1
Source: Nature. 2022 Aug 2;609(7927):611–5. doi: 10.1038/s41586-022-05143-9 (PMC9477737; doi:10.1038/s41586-022-05143-9)
Supplement: Supplementary file 2 — Reporting Summary [file 41586_2022_5143_MOESM2_ESM.pdf]

nature portfolio

Corresponding author(s): Xin Liu, Linfeng Sun

Last updated by author(s): Jul 6, 2022

Reporting Summary

Nature Portfolio wishes to improve the reproducibility of the work that we publish. This form provides structure for consistency and transparency in reporting. For further information on Nature Portfolio policies, see our Editorial Policies and the Editorial Policy Checklist.

Statistics

For all statistical analyses, confirm that the following items are present in the figure legend, table legend, main text, or Methods section.

n/a	Confirmed	
☐	☒	The exact sample size (*n*) for each experimental group/condition, given as a discrete number and unit of measurement
☐	☒	A statement on whether measurements were taken from distinct samples or whether the same sample was measured repeatedly
☐	☒	The statistical test(s) used AND whether they are one- or two-sided<br>*Only common tests should be described solely by name; describe more complex techniques in the Methods section.*
☒	☐	A description of all covariates tested
☐	☒	A description of any assumptions or corrections, such as tests of normality and adjustment for multiple comparisons
☐	☒	A full description of the statistical parameters including central tendency (e.g. means) or other basic estimates (e.g. regression coefficient) AND variation (e.g. standard deviation) or associated estimates of uncertainty (e.g. confidence intervals)
☐	☒	For null hypothesis testing, the test statistic (e.g. *F*, *t*, *r*) with confidence intervals, effect sizes, degrees of freedom and *P* value noted<br>*Give P values as exact values whenever suitable.*
☒	☐	For Bayesian analysis, information on the choice of priors and Markov chain Monte Carlo settings
☒	☐	For hierarchical and complex designs, identification of the appropriate level for tests and full reporting of outcomes
☒	☐	Estimates of effect sizes (e.g. Cohen's *d*, Pearson's *r*), indicating how they were calculated

Our web collection on statistics for biologists contains articles on many of the points above.

Software and code

Policy information about availability of computer code

Data collection	EPU-2.12, SerialEM-3.8.9
Data analysis	RELION 3.1, MotionCor2-1.3.2, CTFFIND4-4.1, cryoSPARC v.3.2.0, ResMap-1.1.5, COOT-0.8.6, PHENIX-1.18rc1-3777, Origin 7.0, Octet Data Analysis software version 9.0, Biacore Insight Evaluation Software Version 3.0.12

For manuscripts utilizing custom algorithms or software that are central to the research but not yet described in published literature, software must be made available to editors and reviewers. We strongly encourage code deposition in a community repository (e.g. GitHub). See the Nature Portfolio guidelines for submitting code & software for further information.

Data

Policy information about availability of data

All manuscripts must include a data availability statement. This statement should provide the following information, where applicable:

- Accession codes, unique identifiers, or web links for publicly available datasets
- A description of any restrictions on data availability
- For clinical datasets or third party data, please ensure that the statement adheres to our policy

The 3D cryo-EM density maps of the apo, IAA-bound, and NPA-bound dimeric state AtPIN1 have been deposited in the Electron Microscopy Data Bank (EMDB, https://www.ebi.ac.uk/emdb/) under the accession number EMD-33691 (https://www.ebi.ac.uk/pdbe/entry/emdb/EMD-33691), 33693 (https://www.ebi.ac.uk/pdbe/entry/emdb/EMD-33693), and 33692 (https://www.ebi.ac.uk/pdbe/entry/emdb/EMD-33692) respectively. Coordinates for the apo, IAA-bound, and NPA-

bound structures model have been deposited in the Protein Data Bank (PDB, https://www.rcsb.org/) under the accession code 7Y9T (https://doi.org/10.2210/pdb7Y9T/pdb), 7Y9V (https://doi.org/10.2210/pdb7Y9V/pdb), and 7Y9U (https://doi.org/10.2210/pdb7Y9U/pdb), respectively. Sequences for the 8 PINs and D6PK in Arabidopsis thaliana are publicly available at Uniprot (https://www.uniprot.org) with the following accession codes: AtPIN1: Q9C6B8, AtPIN2: Q9LU77, AtPIN3: Q9S7Z8, AtPIN4: Q8RWZ6, AtPIN5: Q9FFD0, AtPIN6: Q9SQH6, AtPIN7: Q940Y5, AtPIN8: Q9LFP6, AtD6PK: Q9FG74. Coordinates for the ASBTs and NapA are publicly available at the PDB (https://www.rcsb.org/) with the following accession codes: ASBTNM: 3ZUX, ASBTYF: 4N7W, NapA: 5BZ2. Source data in Figures 1 and 4, and Extended Data Figure 2 are provided as Excel files with this paper.

Human research participants

Policy information about studies involving human research participants and Sex and Gender in Research.

Reporting on sex and gender	*Use the terms sex (biological attribute) and gender (shaped by social and cultural circumstances) carefully in order to avoid confusing both terms. Indicate if findings apply to only one sex or gender; describe whether sex and gender were considered in study design whether sex and/or gender was determined based on self-reporting or assigned and methods used. Provide in the source data disaggregated sex and gender data where this information has been collected, and consent has been obtained for sharing of individual-level data; provide overall numbers in this Reporting Summary. Please state if this information has not been collected. Report sex- and gender-based analyses where performed, justify reasons for lack of sex- and gender-based analysis.*
Population characteristics	*Describe the covariate-relevant population characteristics of the human research participants (e.g. age, genotypic information, past and current diagnosis and treatment categories). If you filled out the behavioural & social sciences study design questions and have nothing to add here, write "See above."*
Recruitment	*Describe how participants were recruited. Outline any potential self-selection bias or other biases that may be present and how these are likely to impact results.*
Ethics oversight	*Identify the organization(s) that approved the study protocol.*

Note that full information on the approval of the study protocol must also be provided in the manuscript.

Field-specific reporting

Please select the one below that is the best fit for your research. If you are not sure, read the appropriate sections before making your selection.

☒ Life sciences ☐ Behavioural & social sciences ☐ Ecological, evolutionary & environmental sciences

For a reference copy of the document with all sections, see nature.com/documents/nr-reporting-summary-flat.pdf

Life sciences study design

All studies must disclose on these points even when the disclosure is negative.

Sample size	All of functional assays were performed with at least three replicates and decribed in the figure legends. The sample size were chosen to ensure the reproducibility of the experiments and to get meaningful results. The sample size were adequate based on distribution of data points and clearly visible effects.
Data exclusions	No data were excluded from the analyses.
Replication	All of functional assays were repeated independently at least three times and all attempts at replication were successful. The number of replications are described in the text.
Randomization	For cryo-EM 3D refinement, all particles were randomly split into two groups. Samples were not randomized for the functional assays as this is not applicable.
Blinding	Blinding was not used in this study, because it is not technically or practically feasible to do so for either the cryo-EM structure determination or the functional assays.

Reporting for specific materials, systems and methods

We require information from authors about some types of materials, experimental systems and methods used in many studies. Here, indicate whether each material, system or method listed is relevant to your study. If you are not sure if a list item applies to your research, read the appropriate section before selecting a response.

Materials & experimental systems

n/a	Involved in the study	
☐	☒	Antibodies
☐	☒	Eukaryotic cell lines
☒	☐	Palaeontology and archaeology
☒	☐	Animals and other organisms
☒	☐	Clinical data
☒	☐	Dual use research of concern

Methods

n/a	Involved in the study	
☒	☐	ChIP-seq
☒	☐	Flow cytometry
☒	☐	MRI-based neuroimaging

Antibodies

Antibodies used

The anti-Myc antibody was purchased from Sigma (Catalog number: M4439, Lot number: 029M4849V, clone 9E10). The anti-Flag antibody was purchased from CoWin Biosciences (Catalog number: CW0287, Lot number: 01222/50503, clone F-tag-01). The synthetic nanobody (Sybody-21) used for structure determination in this study was synthesized using an in vitro translation method as described in the methods section and purified from Escherichia coli. No dilution was performed for purified Sybody-21.

Validation

The anti-Myc antibody and anti-Flag antibody were validated by the commercial vendors:
anti-Myc: https://www.sigmaaldrich.cn/CN/zh/product/sigma/m4439
anti-Flag: https://cwbio.com/goods/index/id/10178
The anti-Flag antibody was also validated by western blot presented in Extended Data Figure 2a, d.
Binding between the Sybody-21 and AtPIN1 is validated by co-migration in gel filtration and clear EM densities in the determined cryo-EM structures.

Eukaryotic cell lines

Policy information about cell lines and Sex and Gender in Research

Cell line source(s)	HEK293F (Sino Biological Inc.)
Authentication	No further authentication was performed for commercially available cell lines.
Mycoplasma contamination	The cell line has been tested negative for mycoplasma contamination.
Commonly misidentified lines (See ICLAC register)	No commonly misidentified cell lines were used in this study.
